# Patient Empowerment for Lifestyle Changes in Metabolic-Bariatric Surgery—A German Nationwide Survey

**DOI:** 10.1007/s11695-025-07989-0

**Published:** 2025-06-23

**Authors:** Alida Finze, Christoph Reissfelder, Susanne Blank, Mirko Otto, Christine Stier, Rafael Meier, Johanna Schreiter, Anastasia Nicolli, Tim Quenstedt, Elias Tigiser, Cui Yang

**Affiliations:** 1 University Medical Centre Mannheim, Mannheim, Germany; 2 Digital Media, DHBW Mannheim, Mannheim, Germany

**Keywords:** Obesity, Bariatric surgery, Physical activity, Patient empowerment, Stigma, Nutrition

## Abstract

**Background:**

Compliance with postoperative follow-up, physical activity, and nutrition is a known problem in patients receiving metabolic-bariatric surgery (MBS). This issue needs to be tackled not only through intervention studies. Patients’ personal preferences and concerns need to be the center of a successful improvement of pre-and postoperative care in MBS.

**Methods:**

A total of 323 participants from support groups all over the country were interviewed via an online survey concerning physical activity, use of media and health-related phone applications, and eating behavior. In addition, 14 open patient interviews were pursued before or after MBS regarding physical activity, eating behavior, and media use.

**Results:**

Of subjects, 64.7% preferred small training groups of 6–10 people. The most popular types of sports were aqua sports, followed by walking and bicycle sports. One-third of the participants stated not enjoying the gym at all. In the open patient interviews, the main reason for this was fear of stigmatization. Sixty-three percent of patients stated a willingness to participate in a sports program designed specifically for MBS patients.

**Conclusions:**

Overall willingness towards an increase in physical activity after MBS is present. However, stigma and pain during sports or the inability to perform certain sports seem to be the main issue keeping patients away from physical activity. We suggest clinical trials towards training protocols in small groups of 6–10 people, aqua, walking, or biking sports, and specialized training protocols for MBS patients.

## Introduction

Metabolic-bariatric surgery (MBS) has become a widespread and currently the most effective treatment for obesity and obesity-related diseases. Alone in 2023, the IFSO registered 480,970 bariatric procedures performed in 24 countries worldwide.

All along this path, patient empowerment and adherence to postoperative follow-up, addressing vitamin deficiencies, preventing sarcopenia, and managing other long-term complications have been a widely discussed matter. Telephone-based interventions [1], app-based follow-up interventions [2], financial incentives [3], and many additional strategies have been subject to research to improve follow-up rates and therefore optimize the long-term postoperative course in MBS patients.

Patient compliance is a central aspect in follow-up, complication management, avoidance of long-term complications, and patient satisfaction. Recent researches have shown that a significant portion of MBS patients are non-compliant regarding postoperative nutritional supplementation [4], postoperative follow-up [5], and overall adherence after surgery [6]. Physical activity adherence before and after MBS also seems to be problematic [7–9].

Physical activity is one of the key components for maintaining a healthy lifestyle and preventing sarcopenia after MBS. Current guidelines recommend an amount of 150 to 300 min of intense physical training per week in post-MBS patients [10, 11]. Post-MBS exercise and exercise protocols are currently subject to multiple clinical trials [12–14]. Next to the improvement of cardiorespiratory fitness and consequently the minimization of cardiovascular risk [15, 16], other factors point to the underrated importance of regular and extensive moderate to intense physical activity (PA) not only before, but also after MBS. Sarcopenia after MBS is a key aspect of physical activity after MBS, as up to 25% of post-MBS patients develop sarcopenia over time [17–19]. There is no data regarding outcomes influenced by post-bariatric sarcopenia in a currently fairly young patient cohort at a later age of 50 to 80 years.

Since the main players are the patients themselves in their own care, it seems logical to look for answers regarding an optimization of pre- and postoperative care within the affected patient group. This study therefore aims to create an overview of patient-centered preferences, optimization concepts, and suggestions made by the affected group concerning pre- and postoperative physical activity in patients with obesity.

## Materials and Methods

### Study Population

In this nationwide prospective study, 323 patients from all German states (excluding Bremen) took part in patient support groups for obesity and MBS. Support groups were contacted through a chairman of “Adipositas Verband Deutschland e.V.” (German Obesity Association). Participation in an online questionnaire was available for two weeks during October 2023. Patients preparing for MBS and patients who had already received MBS at any age were included in the study.

Due to the anonymous online character of this study, ethics approval was not necessary. This study was conducted according to the Helsinki Declaration.

Inclusion criteria for interviews and online questionnaire were informed consent and the ability to read and write in the German language.

### Online Questionnaire

Patients taking part in support groups were contacted via email for participation in an online questionnaire. Participation was voluntary. The questionnaire included baseline data, questions about the use of mobile devices and smart-watches, use of fitness apps, memberships at gyms, participation in fitness programs, preferred types of physical activity, subjective factors for increased motivation concerning physical activity and nutrition and cooking. The online questionnaire was designed to take around 10 minutes. Participation was anonymous. Patients prior to and after MBS were included.

### Patient Interviews

Five female patients were asked to participate in an open interview from one single support group. The patient interviews were conducted anonymously. The patients had all undergone MBS. The interview included personal experience with guidance towards physical activity and nutrition after surgery by their physician, dietary programs, expectations towards physical activity (PA), personal preferences for physical activity, and influence of family, friends, and the support group.

Nine male and female patients were interviewed directly after MBS regarding nutrition prior to and after surgery, physical activity they felt capable of doing, personal preferences regarding physical activity and forms of physical activity, perceived amount of information regarding nutrition, and physical activity as well as experiences with gym visits.

### Data Analysis

Statistical analysis was performed with Microsoft Excel 2021®. Quantitative data analysis was performed for the online questionnaire using frequencies, mean, median, and percentage. Due to the one-stage character of the interviews, further data analysis was not suitable.

## Results

### Baseline Data

A total of 323 patients completed the online questionnaire within the 2 weeks of data acquisition in October 2023. Of the patients, 87.3% were female, and 12.7% of the patients were male. Patient age ranged between 17 and 75 years. Eight patients were aged above 70 years or below 31 years. Further baseline data is presented in Table 1.

**Table 1:** Baseline data

Baseline data	*N* (%)
Male:female	41:282
(%)	(12.7%:87.3%)
Age	
< 18 y	1 (0.3%)
18–30 y	6 (1.9%)
31–40 y	56 (17.3%)
41–50 y	101 (31.3%)
51–60 y	127 (39.3%)
61–70 y	31 (9.6%)
> 70 y	1 (0.3%)
Time relation to surgery	
Not planning surgery	1 (0.3%)
Unsure about surgery	15 (4.6%)
In preparation for surgery	1 (0.3%)
Day of surgery set	45 (13.9%)
< 1 y after surgery	67 (20.7%)
> 1 y after surgery	194 (60.1%)

*y* years. *N* (total) = 323 patients

### Use of Media and Applications

When asked about screentime, 26% of patients reported a mean screentime of 3 h per day. Screentime ranged from 1 h to > 9 h per day; 65% of patients reported a screen time between 2 and 4 h. Ninety-nine percent of patients regularly used their smartphone. Thirty-one of patients stated regular use of a smartwatch device. The patients not regularly using their cellphone either used the computer or a tablet computer instead and were between the age of 51–70 years.

Patients were asked whether they would read an electronic newsletter sent to them regularly regarding their physical activity and nutritional behavior within their MBS therapy. The majority of patients (49%) responded with “yes,” 22% responded that they would possibly read the newsletter, and 29% of patients responded with “no.” The patient cohort was also asked how often they would prefer the newsletter to be sent. The majority (45%) preferred a monthly newsletter in comparison to the option of weekly (25%), two-weekly (26%) information, or a newsletter twice a year (6%).

**Fig. 1:**
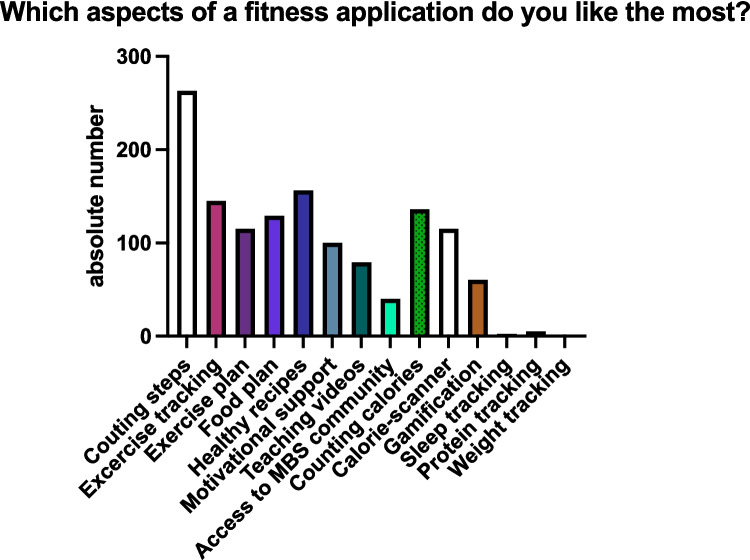
Which aspects of a fitness application do you like the most? Absolute numbers are displayed. More than one answer was allowed

Most patients enjoyed the following functions in fitness applications: counting steps (81.4%), tips about healthy recipes (48.3%), tracking training (44.9%), and counting calories (42.1%). Full results for this item are shown in Fig. 1.

**Fig. 2:**
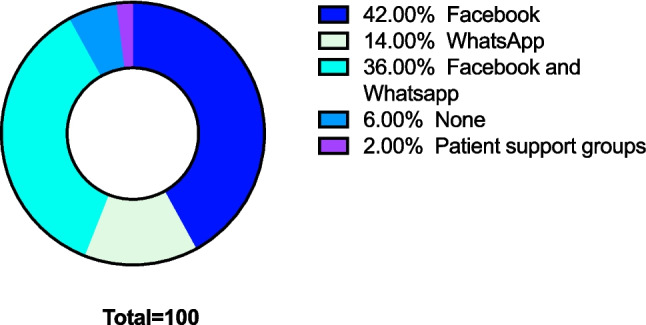
Preferred exchange platforms used in the patient cohort. Pie chart of the preferred exchange platforms in this patient cohort. The chart shows percentages

The patient cohort was also asked about regular exchange platforms they used to contact and speak to other patients. The preferred exchange platforms can be seen in Fig. 2.

### Nutrition and Cooking

When questioned about information towards healthy nutrition, 37% of patients stated the desire for more provided information. Thirty-three percent stated that they did not need more information regarding nutrition, and 30% stated that they were unsure. When asked if the patients were willing to participate in a cooking class with other bariatric patients or patients with obesity or bariatric patients, 64% approved. Only 15% of patients were not willing to participate in a cooking class.

### Physical Activity and Circumstances for Physical Activity

Patients were asked about applications and electronic support for physical activity and fitness. Sixty-eight percent of the patients who completed the online questionnaire stated that they were open to using an online application for physical activity and fitness. Eight percent of patients ruled out the use of this kind of application.

**Fig. 3:**
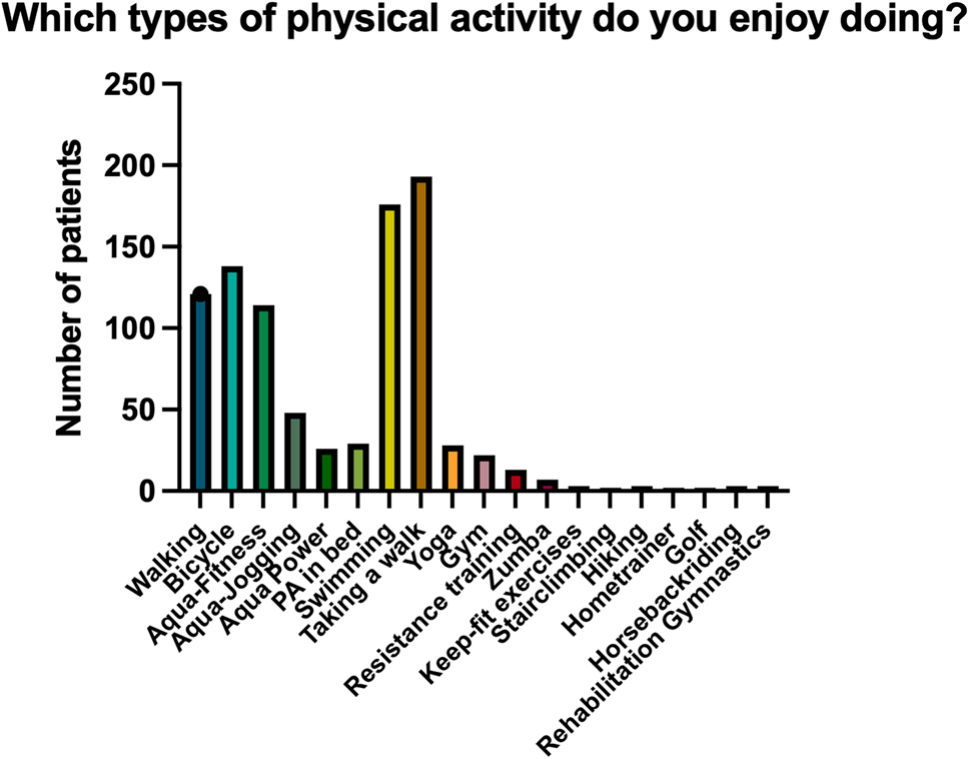
Which types of physical activity do you enjoy doing? Absolute numbers are displayed. More than one answer was allowed

Patients were also asked about the aspect of fitness applications that they perceive as most relevant and helpful. Figure 3 shows the types of preferred activities.

Patients who completed the online questionnaire were consulted about the preferred type of physical activity, the preferred circumstances under which physical activity is performed, and their motivation to take part in physical activity if it was provided to them.

**Fig. 4:**
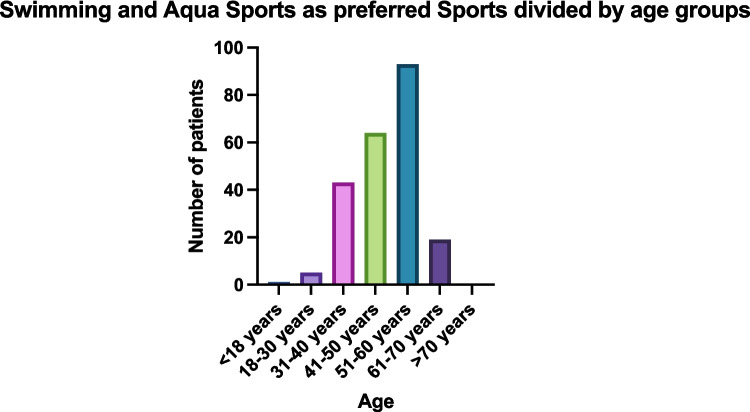
Swimming and Aqua Sports as preferred Sports divided by age groups. Answers are displayed in absolute numbers by age group division. As a comparison, see age group distribution in Table 1

Specifically, the preferred types of physical activity are displayed in Fig. 3. Aqua sports, walking activities, and riding a bicycle were the most preferred types of sports in this patient group. Preferences for types of physical activity were similar in all patient age groups. Exemplarily, this is displayed for aqua sports and swimming in Fig. 4.

A total of 177 (54.8%) patients preferred PA in a group, 107 (33.1%) of patients preferred physical activity on their own. Seventeen (5.2%) of patients stated that they were not open to PA of any type. The rest of the patients either preferred PA with friends or did not have any preference. When questioned about the ideal size of PA groups, most participants preferred a group of 6–10 people (64.7%). When asked specifically about the ideal size of a training group, 7.1% of patients responded that they would prefer PA to be performed on their own.

The participants were asked whether they would participate in a training program designed specifically for them. Sixty-three percent of patients stated that they would participate, 27% that they were unsure, and 10% of patients stated that they would not participate in such training programs.

Overall, 33% of patients claimed to not like going to the gym at all, 25% of participants stated that they somewhat did not like going to the gym or somewhat liked going to the gym, and 16% of participants claimed to like going to the gym very much. The preferred types of sports were similar to the age distribution in the entire patient cohort.

**Fig. 5:**
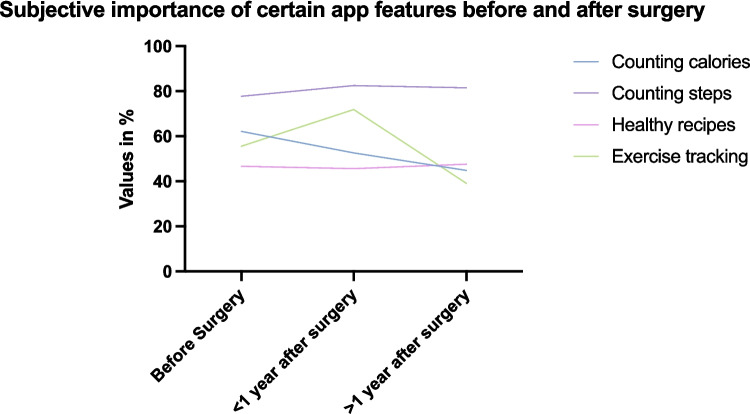
Subjective importance of certain app features before and after surgery. Change of subjective importance of aspects of application functions. Numbers are displayed as percentages. Exercise tracking was found to be subjectively less significant as a smartphone feature in patients that had MBS > 1 year prior

Preferred selective smart phone functions were evaluated over time (before, shortly after, and > 1 year after surgery). Exercise tracking lost relevance > 1 year after surgery. The rest of the preferred aspects did not change in subjective importance. This is displayed in Fig. 5. No differences could be found when comparing age groups regarding types of features preferred in smartphone applications.

### Open Patient Interviews

As described above, 5 patients from a support group who had undergone MBS and 9 patients who had undergone MBS the day before were questioned about their experiences regarding surgery, information about nutrition, and PA and information about PA. These groups were chosen to allow patients to be heard with most of the excess weight existing as well as after weight loss. This ensures that patients are heard in a vulnerable phase directly after surgery when starting physical activity is a hurdle that needs to be taken. This qualitative data showed that most of the patients complained about pain and lack of mobility during most sports. Aqua sports and bicycle training were mentioned as types of PA that could be performed easily and with little pain. The interviewed patients had the desire for increased information flow about physical activity and nutrition through the attending physicians. Some patients suggested specialized training groups for MBS patients. Multiple patients stated that they did not visit gym facilities but might visit them if their health insurance covered the costs.

Regarding the given reasons for lack of PA, some patients stated that they were not willing to adhere to physical activity because they do not like PA or did not feel the motivation for PA. Other patients stated that they did not like going to the gym because they felt stigmatized.

Regarding nutrition, the majority of patients described an eye-opening effect during the obligatory consultations with nutritionists prior to surgery. They stated that they had undergone many diets but then had a weight rebound after some time. Many of the patients stated that they did not know much about balanced and healthy nutrition prior to consulting nutritionists before MBS.

## Conclusion

Obesity as a chronic disease needs to be treated with a multimodal approach, and adequate conservative treatment should be performed before and after MBS. These interventions include a psychological evaluation [20], exercise [21, 22], and consultation with a dietician [21, 22]. Preoperative lifestyle intervention is an essential part of MBS and facilitates the patients’ postoperative course not only regarding weight outcome, but also regarding the recurrence of unhealthy lifestyle behavior.

However, recommendations towards lifestyle and behavior before and after surgery cannot be effective if they do not match patients’ needs and preferences. This is one of the reasons that lifestyle interventions are effective at first, but do not have a lasting effect over more than 1–2 years [23]. Results for long-term weight loss and weight loss maintenance via lifestyle interventions only over more than 1 year are very poor [24, 25].

It is unfathomed that patient empowerment and adequate information seem to be key to a change in lifestyle for MBS patients. Successful MBS is also linked to lifestyle change regarding food, supplementation, and physical activity. This has become clear in the past years through the effects of MBS such as malnutrition [26–29], vitamin deficiencies [30], and sarcopenia [19, 31, 32]. Since the patient is the main character within MBS, the patient is responsible for adequate PA and nutrition in the end. The study acknowledges the difficulties patients face in changing long-established habits, highlighting the need for ongoing support and guidance.

This study, which included a large cohort from all parts of Germany, was able to create a good insight into detailed information regarding the improvement toward lifestyle interventions in MBS patients. It explores patients’ attitudes and preferences regarding physical activity and nutrition in the context of metabolic-bariatric surgery. The information gained from this survey builds the base for multimodal postoperative MBS care. Good post-MBS care is not possible without patient inclusion.

While patients stated a willingness to participate in fitness programs and PA classes in the online survey, this is not reflected entirely in the open patient interviews, in which some patients showed reluctance due to lack of motivation. The preferred types of PA seem to be aqua sports, walking sports, and bicycle training. However, the gym does not seem to be a preferred environment for PA for this specific patient group. Patients preferred group sports in small groups of 6–10 people. Almost one-third of patients stated that they prefer to perform PA among other bariatric participants, suggesting that patients may feel more comfortable and less stigmatized in such settings. When the participants were asked about the ideal group size for PA, however, only a small percentage (7.1%) of patients still preferred PA on their own. This was reflected in the qualitative patient interviews. The interviewed patient group stated great fear of stigma when going to the gym or taking part in large training groups. This also aligns with results from previous studies [33–35] and was taken into account in the new German guidelines for obesity [36].

It should not be left out of account that stigma also plays a great role in sports in a patient group with obesity that is not as physically fit as the average person [33, 37]. If MBS patients feel uncomfortable in an environment in which they need to come close to their limits of physical exertion, it can be assumed that adherence and willingness to PA decrease significantly.

Nutritional management and nutritional guidance seem to be further central aspects for the patients that participated in this survey. Sixty-three percent of patients stated that they do not need more information about nutrition or are not sure if they need more information about nutrition currently. Only 37% of patients felt that they were missing information about nutrition after and before MBS. Consultation with a nutritionist seems to have a long-lasting effect on MBS patients since patients described this consultation as eye-opening regarding their nutritional habits and in most cases did not feel the need for more consultation in the survey. In accordance with the known positive effects of additional nutritional consultations for MBS patients in terms of reduced postoperative nutritional complication rates and lower readmission rates [38–40], a similar effect might be postulated regarding education on PA.

The data presented from this survey suggests that subjective needs and preferred types of motivators and sports do not change significantly with different age groups or during the course of surgery. This survey was cross-sectional and therefore does not represent change over time adequately. However, it does raise the question if needs regarding nutrition, PA, and especially support for nutrition and PA differ between different age groups and within the course of bariatric treatment. This should be explored more closely in future studies.

A specialist consultation regarding nutrition can have an effect on the perception of support for patients. This effect remains visible many years after MBS. The desire for increased information flow regarding PA and sports for MBS patients should therefore be taken as a starting point. Consultation with fitness and sports specialists should be considered an essential part of preparation for MBS and post-MBS treatment. Similar to the subjective perception regarding information about nutrition, this kind of intervention might have the same effect for PA. We expect a potentiated effect from the combination of PA and adequate nutrition towards muscle preservation, as data from healthy and MBS patient groups suggest [41, 42].

Based on the findings of our study, we give several practice-related recommendations.

- Patients would like to be supported with fitness and sports specialist consultations before and after surgery. Currently, several training protocols and programs are being evaluated within clinical trials [13, 14].
- For a successful application of PA training programs, training protocols should include low-impact activities like aqua sports, cycling, and walking in small groups of 6–10 participants [43].
- Environment to minimize stigma and maximize comfort for MBS patients should be created.
- If possible, patients should be provided with different training options.
- Combining nutrition and PA interventions on muscle maintenance and general health outcomes could yield significant benefits.
- Finding an approach towards more physical activity should also include education about limiting sedentary behavior (e.g., climbing stairs instead of using the elevator etc.)

A clear limitation of this study is a possible selection bias with mainly female participants in the survey. There is no data regarding actual physical activity or data that verifies patients’ statements towards PA and eating behavior. This study can therefore only give a subjective point of view on improvements regarding postoperative patient empowerment. In addition, the patients included in this survey were already part of support groups. Patients not being supported by other patients or support group leaders might encounter greater challenges in adhering to recommendations or might have different needs from those displayed in this survey.

Concluding, treatment of MBS patients after surgery needs to be seen in a more holistic way. To achieve this goal, we need to find out more about how to engage patients more properly in their postoperative care and follow-up. With this survey, we were able to deliver essential pieces of information to open new pathways towards implementation of actual fitness goals in bariatric patients and patients with obesity. Adherence and motivation in the MBS patient group have been a great hurdle towards adequate treatment and follow-up[4, 6]. Patients’ preferences towards sports and an appreciative environment when performing physical activity need to be taken into account when tackling physical fitness and sarcopenia after MBS. To increase trust and adherence with physicians, concerns about stigma, nutrition, and physical activity need to be taken seriously. Maximizing the percentage of physical activity and reducing sedentary behavior within the 24-h movement approach might be a helpful entry point.

## Data Availability

No datasets were generated or analysed during the current study.
